# Microbiota and seminal quality: A systematic review

**DOI:** 10.5935/1518-0557.20230008

**Published:** 2023

**Authors:** Isadora de Almeida Gomes, Paula Bruno Monteiro, Gabriel Acácio de Moura, Nayara Oliveira Santos, Cristina Tonin Beneli Fontanezi, Sabrina Vieira de Souza, Clara Andrade Teixeira

**Affiliations:** 1 Graduating in biomedicine at the Christus University Center - UNICHRISTUS, Fortaleza, CE, Brazil; 2 Master in Public Health from the Federal University of Ceará - UFC, Fortaleza, CE, Brazil; 3 Master in Veterinary Sciences from the State University of Ceará - UECE, Fortaleza, CE, Brazil; 4 Master in Medical Microbiology from the Federal University of Ceará - UFC, Fortaleza, CE, Brazil; 5 PhD in Experimental Pathology from the Faculty of Medicine of Ribeirão Preto da University of São Paulo (FMRP/USP), Ribeirão Preto, SP, Brazil

**Keywords:** microbiota, male infertility, seminal quality

## Abstract

The microbiota is composed of numerous resident microorganisms, which contribute to the health and illness of the individual. When the microbiota is in dysbiosis, it can cause some pathological processes and in men it can be correlated with male infertility, so the present study does a systematic review, identifying whether there is a correlation between the microbiota and seminal quality. We analyzed 7 papers published in PubMed, Medline and the Cochrane library databases, in English and published between 2012 and 2022. In men with normal semen parameters, a higher prevalence of Lactobacillus. There was a higher prevalence of *Prevotella* in patients who had some seminal alteration. We conclude that the microbiota is correlated with seminal quality, since the decrease in Lactobacillus and the increase in other species is seen in infertile men.

## INTRODUCTION

Currently, studies on the microbiota have been widely carried out in several parts of the world, since the microbiota consists of the abundance of microorganisms, especially bacteria from which the human body is colonized, and has an influence on the health and disease of the host, being also known as the “second human genome” (Grice & Segre, 2012).

However, when talking about microorganisms, most of the time it is correlated with pathological processes; however, numerous reports in the literature demonstrate the benefit of the microbiota for the well-being of the individual, since the imbalance of the normal microbiota can favor the growth and appearance of pathogenic microorganisms. An example of this is the *Lactobacillus*, which are part of the intestinal microbiota, and can be used to treat some diseases, such as to alleviate the characteristic symptoms of irritable bowel syndrome, leading to a reduction in abdominal pain and improvement in quality of life (Preston *et al*., 2018). Another example is the study by Soleimani *et al*. (2017), which concluded that the use of probiotics containing some *Lactobacillus* species had significant results in decreasing fasting glucose levels after 12 weeks of use in diabetic patients.

Therefore, a dysbiosis can lead to the emergence of diseases and damage to health. There are studies correlating the presence of some pathogens, whether bacteria, fungi or protozoa, which can result in damage to male fertility in several ways; among them alterations in seminal quality and inflammation of the epididymis and testes (epididymitis and orchitis respectively). Furthermore, some microorganisms can even cause obstructions in the reproductive tract (Gimenes *et al*., 2014; Shang *et al*., 2014).

Thus, a major problem arising from changes in the male microbiota is epididymitis, which is among one of the main causes of male infertility and can affect men of all ages. The main causes of acute epididymitis, in about 50% of cases, are *Escherichia coli, Chlamydia trachomatis* and *Neisseiria gonorrhea*. This inflammation, in the long term, can result in irreversible damage, even after treatment. The damage may be due to characteristics of the immune system itself, which releases cytokines and chemokines, leading to chronic inflammation of the epididymis, causing cell proliferation or scarring in the male genital tract (Zhao *et al*., 2020).

### Male infertility and the microbiota

Infertility is when a couple is unable to conceive within a year, having frequent sexual intercourse, without the use of contraceptives (WHO, 2020). The Brazilian Society of Assisted Reproduction (Carneiro, 2017) reports that 30% of infertility cases come exclusively from men. In Latin America, until 2015, almost half of the couples who gave birth through assisted reproduction techniques used the Intracytoplasmic Sperm Injection (ICSI) technique, which is indicated in cases of low semen quality.

Male infertility can be caused by several factors, congenital or acquired, endocrine disorders, autoimmune and systemic diseases, lifestyle habits, urogenital abnormalities and infections of the urogenital tract. However, in up to 30-40% of cases of male infertility, it is idiopathic, that is, when the reason is not known (Jungwirth *et al*., 2019).

It was discovered that in healthy fertile men, in addition to the semen having proteins, enzymes, lipids and other substances, there may also be the presence of a variety of microorganisms such as *Lactobacillus* and *Prevotella* at low concentrations (Altmäe *et al*., 2019). The microbiological analysis of semen is performed when the individual has leukocytospermia (increased number of leukocytes), presence of numerous bacteria in the semen and presence of symptoms, using culture on microbiology plates or gene sequencing for identification (Andrade-Rocha, 2008). The sequencing of the 16s rRNA gene has been increasingly important and necessary for the identification of difficult-to-identify bacteria that in turn may be correlated with asymptomatic infections and infertility (Watts *et al*., 2017).

Thus, it would be of great importance to evaluate the microbiota of individuals with alterations in seminal quality, since men considered infertile due to idiopathic causes may actually have a microbiota with microorganisms that favor the reduction of seminal quality and, if such a relationship is proven, the treatment of the patient could be very specific and he could eventually become fertile again; therefore, the objective of the present study was to identify, through a literature review, the correlation between microorganisms and seminal quality.

## MATERIALS AND METHODS

This is a systematic review, with the objective of reporting a correlation between the microorganisms and seminal quality, and reporting microorganisms in infertile patients. It was used as a guide for the preparation of this study the precepts of the declaration “Preferred Reporting Items for Systematic Reviews and Meta-analysis” (PRISMA).

### Search strategy

Data collection started in August 2021 and lasted until March 2022.

PubMed, Medline and Cochrane library scientific bases were used, adopting as keywords: “microbiota or microbiome”, “16S” and “semen or sperm”. Articles published between 2012 and March 2022, in English, were evaluated.

### Article selection criteria for review

Articles published between 2012 and 2022 were included, which were complete and originals, in the English language and that report the seminal quality correlated with the microbiota. Unavailable articles, animal studies and review articles were excluded.

### Measurements

After screening the articles using the inclusion and exclusion criteria, we read the articles to analyze the papers that would compose this study. From this we tabulated: the number of patients included in the study, the classification according to seminal quality (men with normal semen parameters, men with idiopathic infertility, men with asthenozoospermia, men with azoospermia, and men with oligospermia), the microorganisms present and the methodology used.

## RESULTS

**Figure 1:**
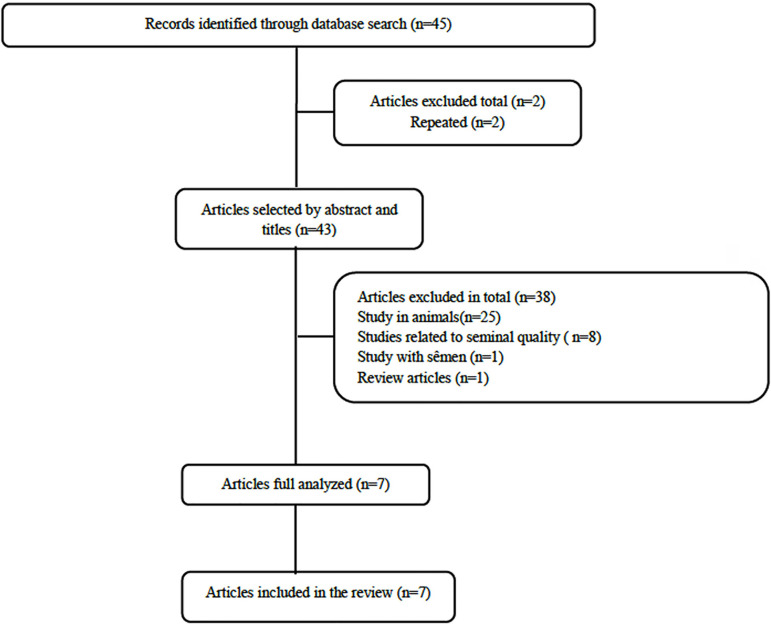
Flowchart detailing the selection of studies for inclusion in the review. PRISM.

We found 45 papers using the PubMed (n=36), Medline (n=7) and Cochrane library (n=2) databases; however, 2 papers were excluded due to duplicity. During the screening, 34 papers were excluded after reading the title and abstract. There remained 9 papers, which were read in full, resulting in a total of 7 papers for analysis (Figure 1).

Each study was relevant for writing this study as shown in Table 1. All authors used the sequencing of the 16 S rRNA gene as a way of identifying microorganisms present in semen. Only Lundy *et al*. (2021) used shotgun sequencing together. Hou *et al*. (2013) used, in addition to sequencing the 16S gene, the Gram stain methodology in semen, where they observed that the number of bacteria was greater than the number of sperm in most samples of sperm donors and that the amount and bacterial morphology were highly variable among individuals.

**Table 1:** Microbiota assessment depending on seminal quality. Normospermia (normal semen parameters), oligoasternospermia (low concentration and low sperm motility), asternosperm (low sperm motility), azoospermia (absence of sperm in the ejaculate), oligospermia (low concentration of sperm) and leukocytospermia (increase in the number of leukocytes in semen.

Author	Year	Number of individuals	Classification according to seminal quality	Microorganisms present in each group	Methodology
**Hou *et al*.**	2013	77	Healthy sperm donors	*Streptococcus; Corynebacterium; Finegoldia; Veillonella; Lactobacillus; Prevotella; Staphylococcus; Anaerococcus; Peptoniphilus*	16 S rRNA gene sequencing and Gram stain
			Asthenospermia	*Prevotella; Peptoniphilus; Lactobacilus; Clostridiales*	
			Oligoasthenospermia	*Corynebacterium; Staphylococcus; Finegoldia; Anaerococcus*	
			Oligoasthenozoospermia and severe azoospermia	*Ralstonia; Prevotella; Aerococcus; Gemella*	
**Baud *et al*.**	2019	94	Normal spermogram parameters	*Staphylococcus; Lactobacillus*	16 S rRNA gene sequencing
			One or more abnormal parameters	*Prevotella*	
**Yang *et al*.**	2020	159	Control men	*Acinetobacter; Pelomonas; Streptococcus; Lactobacillus*	16 S rRNA gene sequencing
			Oligoasthenospermia	*Ralstonia; Bacteriodes; Prevotella; Lactobacillus*	
			Asthenospermia	*Sneathia; Ralstonia; Ureaplasma; Aerococcus; Anaerococcus; Corynebacterium*	
			Azoospemia	-	
			Oligospermia	-	
**Yao *et al*.**	2022	87	control men	*Lactobacillus* ↑; *Streptococcus* ↓	16 S rRNA gene sequencing
			Asthenospermia		
			Leukocytospermia	*Streptococcus* ↑	
			Asthenospermia and WBC count >1x106ml	*Lactobacillus* ↓	
**Lundy *et al*.**	2021	37	Control men	*Collinsella* ↑; *Pseudomonas* ↓	16 S rRNA gene sequencing and shotgun sequencing
			Men with idiopathic infertility	*Aerococcus* ↑*; Gardnerella; Prevotella; Pseudomonas; Collinsella* ↓	
**Puerta Suárez & Cardona Maya**	2021	10	Group control	*Rhizobiaceae; Burkholderia; Achromobacter; Delftia; Campylobacter; Ezakiella; Anaerococcus; Prevotella; Haemophilus*	16 S rRNA gene sequencing
			Prostatitis group		
**Okwelogu** ***et al*.**	2021	36	Normospermic men	*Lactobacillus* ↑*; Gardnerella; Veillonella; Corynebacterium; Escherichia coli; Hemophilus; Prevotella*	16 S rRNA gene sequencing
			Men with oligospermia	*Prevotella* ↑*; Escherichia coli;* *Lactobacillus; Gardnerella*	

In total, 500 individuals were analyzed in the present study. Diverse types of separation were performed between the participants, all articles had the control group (normospermics). In about 40% of the studies, there was a separation from the group of men with asthenospermia (Hou *et al*., 2013; Yang *et al*., 2020; Yao *et al*., 2022); whereas 30% of the studies had groups of men with oligoasternospermia (Hou *et al*., 2013; Yang *et al*., 2020), azoospermia, oligospermia and leucospermia (Yang *et al*., 2020; Okwelogu *et al*., 2021).

**Figure 2:**
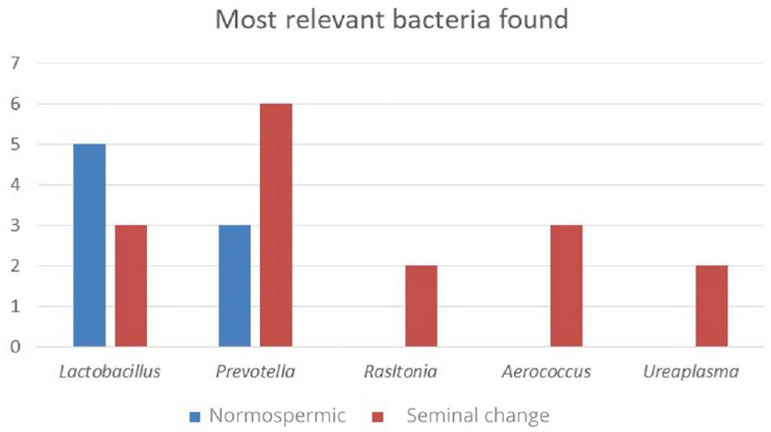
Presentation of the bacteria that showed relevance because they were present in two or more studies in normospermic men and in men who had some type of alteration in seminal quality.

As we can see in Figure 2, in 5 studies, individuals considered normospermic had a prevalence of *Lactobacillus* genus, in most cases, confirming its presence in greater abundance compared to other bacteria. In patients with seminal alterations, *Lactobacillus* was also found; however, they were reduced.

On the other hand, 6 studies found *Prevotella* in individuals who had some type of seminal alteration. Although 3 studies reported the presence of *Prevotella* in normospermic patients, its concentration was low. Finally, genus *Rasltonia, Aerococcus* and *Ureaplasma* were only found in men who had some type of change in seminal quality.

## DISCUSSION

Male infertility has been increasingly frequent, environmental factors and lifestyle habits can have an unfavorable impact on male fertility, such as changes in the microbiota. These changes can lead to symptomatic or asymptomatic infections, which can cause changes in spermatogenesis or induce toxin production. In addition, they produce oxidative stress, which impairs seminal quality and can cause infertility (Katz *et al*., 2017; Oghbaei *et al*., 2020) in the partner, since the couple’s microbiota are influenced by both (Pasqualotto, 2007).

Understanding the significant importance of microbiota analysis, the advancement of identification technologies has become increasingly relevant, such as the use of bacterial genetic sequencing technology, which allows the identification of little-known and difficult-to-identify pathogens such as *Ralstonia*, which was observed in two studies analyzed by the present study. *Ralstonia* was present in patients with oligoasthenospermia and severe azoospermia. These are aerobic, non-fermenting Gram-negative bacilli and there are three species (*Ralstonia pickettii, Ralstonia insidiosa* and *Ralstonia mannitolilytica*) present in water and soil. In addition to harboring former members of the *Burkholderia spp*. (*Burkholderia pickettii* and *Burkholderia solanacearum*) (Ryan & Adley, 2014).

The literature reveals that *Ralstonia* is an opportunistic bacterium and its description in reproductive tract infection is extremely rare, a case report showed that the patient had semen with normal parameters regarding motility, progressively motile sperm count, viability and morphology; however, she did not have a successful pregnancy after a year of trying. The presence of large masses of globular debris, consisting of both peroxidase-positive cellular structures and acellular debris, was noteworthy. The presence of *Ralstonia pickettii* was then identified, after which antibiotic treatment was performed, which resulted in a considerable decrease in peroxidase-positive cell (Carrell *et al*., 2003). Another bacterium that has relevance to seminal quality is *Aerococcus*, which was seen in the semen of men with asthenospermia, idiopathic infertility, oligoasternospermia and severe azoospermia. It refers to Gram-positive cocci, facultative anaerobes and some species may be part of the microbiota of both humans and animals. Two genera are most relevant: *Aerococcus urinae* and *Aerococcus sanguinicola*, responsible for causing genitourinary tract infections, endocarditis and sepsis (Rasmussen, 2016). *Aerococcus urinae* is more associated with urinary infections in elderly men, although its presence in the female microbiota is associated with normality (Senneby *et al*., 2012). Its presence in the microbiota of men with seminal alterations may be correlated with the ability of this bacterium to form a biofilm, since in addition to being an escape mechanism against the antibiotic, it may obstruct the ducts through which spermatozoa pass (Shannon *et al*., 2010).

Another pathogen that aroused interest in the results was the bacterium *Ureaplasma*, which is part of the *Mycoplasma* family, this bacterium has a characteristic of not having a cell wall around its cell membrane, in most cases it is present in the normal microbiota and when it causes infection, it is usually asymptomatic and most commonly associated with urinary tract infections (Al-Sweih *et al*., 2012). Oliveira *et al*. (2014) concluded that the presence of *Ureaplasma* in semen and its impacts on fertility are still not well-elucidated, requiring further study focused on the mechanisms of this microorganism, since its presence is observed in infertile men.

*Staphylococcus* is another genus that has shown relevance, since they are cocci, usually seen grouped in “grape bunches”, Gram-positive that are naturally resident in the human microbiota (Taylor & Unakal, 2022). A study carried out in Colombia with the objective of determining the effect of soluble bacterial factors of *Staphylococcus aureus, Staphylococcus capitis* and *Staphylococcus epidermidis* on conventional and functional seminal parameters, used 20 samples from donors with normal sperm parameters. After incubation of the spermatozoa, separately with the soluble factors produced by the metabolism of each bacterium, there was a 25% deterioration in motility in 15 minutes in the spermatozoa submitted to the factors of metabolism of the *Staphylococcus aureus* sensitive to oxacillin, already in resistant strains with oxacillin, the negative effect on motility was immediate. The oxacillin-sensitive *Staphylococcus capitis* strains showed a decrease in sperm motility by 30% in 30 minutes. Finally, sperm subjected to the soluble factors of the oxacillin-resistant strain of *Staphylococcus epidermidis* had a 30% reduction in motility within 15 minutes. Thus, the study shows that the factors produced by *Staphylococcus* interact with sperm and decrease seminal quality (Pardo *et al*., 2015).

In addition, the *Staphylococcus hemolytic* species was the focus of a study with 80 infertile patients, 94% with primary infertility and 6% with secondary infertility. Of the cases of primary infertility, 79% had the presence of *Staphylococcus hemolytic* and its presence may be related to structural changes and sperm motility (Al-Ghizzawi & Jomaa, 2018).

*Prevotella* was one of the bacteria most highlighted in the results, as it was very much present in men with some type of seminal alteration. It is a Gram-negative, anaerobic and immobile bacillus (Shah & Collins, 1990). Currently, studies show that its presence, in high concentration, is associated with abnormal spermogram groups (Gachet *et al*., 2022). This elevation may result from changes in the microbiota of other systems of the human body and also cause dysbiosis in semen. An unbalanced, high-fat diet can cause an imbalance in the gut microbiota, for example. In an experimental study with mice divided into two groups, in which one received an adequate diet and the other group a high-fat diet, the researchers concluded that the mice on the high-fat diet had weight gain, as well as a chronic inflammation due to bacteria. When they examined the semen of these rats, they observed a correlation with infertility, as they showed an abundance of *Prevotella* with a significant decrease in motility and sperm count. Interestingly, intestinal inflammation precedes a weakening in sperm production and maturation (Ding *et al*., 2020). In addition, tests carried out with zebrafish showed that a high-fat diet alters testicular microbiota. In an experiment, there was a drastic decrease in sperm motility in obese fish, leading to believe that it is related to the change in testicular microbiota (Su *et al*., 2021).

On the other hand, the presence of *Lactobacillus* demonstrates a positive relationship with seminal quality, since researchers carried out a study with 96 samples, and when analyzing the quality of the semen concluded that *Lactobacillus* crispatus have positive effects on quality, sperm concentration and in maintaining the normal microbiota, protecting against the negative influences of other bacteria such as *Prevotella*, Hemophilus and *Pseudomona* (Weng *et al*., 2014). They are Gram-positive, facultative anaerobes, which are present in greater amounts in the intestine and in the vaginal microbiome (Oliveira *et al*., 2017). In the vaginal microbiota, its correlation with protection against infections is already well established; when there is a balance between the 12 resident microorganisms, especially *Lactobacillus*, due to their ability to provide a low pH, by releasing lactic acid, making the colonization of other bacteria difficult, in addition to releasing hydrogen peroxide that inhibits the exacerbated growth of pathogens (Mirmonsef *et al*., 2011).

Voroshilina *et al*. (2021) used 227 normospermic semen samples and concluded that in half of the cases they had a predominance of *Lactobacillus, Enterobacteriaceae spp.* and *Enterococcus spp*. In a study where they used 46 male partners of infertile couples and were separated into two groups, where 20 received the active drug (Flortec) and 21 received starch. After 6 months, the group that used the drug had improvements in ejaculate volume, sperm concentration, sperm motility, number of ejaculated sperm and percentage of typical forms, while the group that used starch showed no improvement in seminal parameters, finally, 5 of the 20 patients treated with the active drug had a child, while no patients treated with the control substance had children (Maretti & Cavallini, 2017). In a study with obese rats, they concluded that the use of probiotics with *Lactobacillus rhamnosus* PB01 (DSM 14870) acted on the parameters of spermatic velocity and movement, which may be associated with the direct effect of probiotics on spermatogenesis and maturation process or indirectly, removing the adverse effects of obesity and increasing the level of total antioxidant capacity (Dardmeh *et al*., 2017).

Thus, it is worth highlighting that the presence of Gram-negative bacteria such as *Ralstonia* and *Prevotella* produce bacterial lipopolysaccharide, which is an endotoxin present in the cell wall of Gram-negative bacteria. This was evidenced in the study carried out by Li *et al*. (2016), who used healthy men between 25-35 years old and with a reproductive history of the last two years to evaluate the *in vitro* toxicity of bacterial lipopolysaccharide, the result of the study was that this endotoxin inhibits sperm motility by reducing intracellular concentrations of cyclic adenosine monophosphate.

Another interesting study was that of Sahnoun *et al*. (2017) who analyzed seminal samples from 73 patients to investigate a cause of infertility; and they identified that human spermatozoa have the Toll-like receptor 4, which reacts to bacterial lipopolysaccharide stimulation and results in production of reactive oxygen species. Oxidative stress results from a disproportion between the formation of reactive oxygen species and the antioxidation system. Thus, reactive oxygen species are responsible for generating male infertility, causing structural changes to the sperm head and its intermediate membrane, leading to a reduction in sperm motility, in addition to causing sperm DNA fragmentation (Walczak-Jedrzejowska *et al*., 2013).

## CONCLUSION

As a result, we can conclude that there is a divergence between the microbiota of fertile and infertile men. In infertile men there was a greater presence *of Ralstonia, Prevotella, Ureaplasma, Staphylococcus* and *Aerococcus*. In men with good seminal quality, the prevalence was *Lactobacillus*. Although it is possible to observe this difference, there was a high variation between the populations of the analyzed articles, since the microbiota is interfered by several causes. The identifications of microorganisms present in the semen show great relevance, since the treatment is directed and as described, there are reports where infertile men become fertile again after the correct treatment. Finally, further studies are needed with a greater number of samples and a more homogeneous population in order to reduce variations and achieve a correlation between microorganisms and more reliable seminal quality.
